# Prioritizing limb loading improves symmetry during dual-tasking in individuals following anterior cruciate ligament reconstruction

**DOI:** 10.3389/fspor.2023.1090694

**Published:** 2023-02-28

**Authors:** Ming-Sheng Chan, Susan Sigward

**Affiliations:** ^1^ Performance Science, San Francisco Giants, San Francisco, CA, United States; ^2^ Division of Biokinesiology and Physical Therapy, University of Southern California, Los Angeles, CA, United States

**Keywords:** ACLR, dual-task, limb loading symmetry, automaticity, rehabilitation

## Abstract

Understanding the extent to which attention prioritization interfere with limb loading in daily activities following anterior cruciate ligament reconstruction (ACLr) is important for reshaping loading behaviors. A dual-task paradigm, prioritizing limb loading symmetry (LLS) during standing or response time during an upper extremity task response time task was used to probe the effects of attention prioritization of loading. Individuals 115.6 ± 17.8 days post-ACLr (ACLr; *n* = 13) and matched healthy individuals (*n* = 13; CTRL) performed a simple response time (RT) task and 2 dual tasks prioritizing limb loading (LS-RT) and response time (RT-LS). 2 × 3 General Linear Model repeated measures analyses determined effects of group and focus condition on LLS error and response time. Significant interaction (*P* = 0.010) was noted in LLS error. ACLr group, exhibited greater LLS error in RT (*P* = 0.001) and RT-LS (*P* = 0.001) than LS-RT condition. ACLr group exhibited greater LLS error in the RT (*P* = 0.001) and RT-LS (*P* = 0.040) than CTRL, but not in LS-RT. A main effect of condition (*P* < 0.001) for response time indicated that times were slower in LS-RT compared to RT (*P* < 0.001) and to RT-LS (*P* < 0.001) for both groups. These data suggest that limb loading symmetry during standing is more automatic for controls than individuals following ACLr. Unlike controls, improving loading symmetry during standing requires additional attention in individuals in early recovery following ACLr.

## Introduction

Biomechanical studies have shown that individuals following ACLr adopt loading strategies that shift mechanical demand away from the surgical knee and limb during functional and athletic tasks (1–6). These strategies are most apparent during bilateral tasks that require equal distribution of weight across both limbs. A recent study found that at 3 months post-ACLr, individuals underloaded their surgical limb during standing, sit-to-stand and squat tasks by as much as 24% when they were not specifically attending to task performance (7). Despite specific emphasis on increasing loading of the surgical limb during postoperative rehabilitation, these deficits appear to persist over time. Longitudinal assessments of loading indicate that asymmetrical loading observed during squatting at 3 months post-ACLr does not improve at 5 months (8). It is suggested that traditional rehabilitation is not sufficient for the restoration of limb and joint loading. When considered along with studies that report similar deficits during squatting in individuals up to 22 months post-surgery (3) and during landing 2–3.5 years post-surgery (6, 9), it is clear that these early underloading strategies persist long-term. The persistence of a generalized asymmetrical loading strategy is of concern as a prospective assessment of athletes following ACLr found that the odds of suffering a second ACL injury were 2.3 times greater in those who exhibited asymmetrical knee loading during a drop land at the time they returned to sports (10). Asymmetrical ground reaction forces during landing have been prospectively linked to risk for ACL injury in healthy individuals (9). In addition, asymmetrical loading has been attributed to the progression of knee osteoarthritis (11, 12).

There is evidence to suggest that asymmetrical loading strategies observed in early rehabilitation are not the consequence of an inability to accommodate loading demands, but a strategy carried over from early adaptations to joint level impairments experienced following injury and surgery. Individuals 3 months post-ACLr are able to improve loading symmetry by up to 14% during standing, sit-to-stand and squatting tasks when they were instructed to focus on distributing loads evenly through the limbs (7). These improvements from natural loading to instructed loading conditions suggest that increasing loading of the surgical limb during functional tasks may require additional attention at this time post-surgery.

Evaluations of individuals 1-year post-ACLr that show greater cortical activation during motor accuracy tasks and increased postural errors in dual-task conditions, support this premise. When compared to healthy controls, greater cortical activation (electroencephalogram data) was observed during a quadriceps force reproduction task in individuals 12.0 ± 4.7 months post-ACLr in order to achieve the same accuracy (13). The authors suggest that individuals following ACLr require more focused attention to accomplish the same motor task involving the knee joint than healthy individuals. Moreover, the introduction of a cognitive task during a single-limb balance task increased balance errors in individuals 14 months post-ACLr compared to controls (14–17).

Currently, it is not known if maintaining limb loading symmetry during common daily activities requires additional attentional resources for individuals following ACLr. If individuals require more cognitive resource to achieve appropriate loading in early rehabilitation, one might expect that the effects of exercises performed in rehabilitation may not carry over to daily activities with different attentional prioritizations (18, 19). Understanding the extent to which attention prioritization interferes with loading in a common daily activity in early rehabilitation is important for reshaping early loading behaviors.

Therefore, the purpose of this study was to use a dual-task experimental paradigm to determine the effects of attention prioritization of maintaining limb loading symmetry; comparing the performance of an upper extremity response time task and the degree of loading symmetry under conditions with different attentional prioritizations. It is hypothesized that during the dual-task condition that involves two performance goals (loading symmetry and response time), improved limb loading symmetry and increased response time will be observed in the condition where attention is prioritized to loading symmetry but not in the condition where attention is prioritized to response time in individuals following ACLr; however, these tradeoffs between loading symmetry and response time will not be observed in healthy individuals.

## Materials and methods

Two groups of participated in this study: individuals 115.6 (17.8) days post anterior cruciate ligament reconstruction (ACLr; *n* = 13) and healthy controls (CTRL; *n* = 13). Participants’ descriptive information is reported in Table 1. The participants in the ACLr group were recruited from four physical therapy clinics in the greater Los Angeles area. They were enrolled in the study if they were (1) between the ages of 14–50, (2) 10–16 weeks status post ACLr, (3) currently participating in physical therapy, and (4) cleared to perform the experimental tasks. Participants in the control group were recruited to match the participants in the ACLr group based on age- (±2 years), sex-, height-, weight-, and physical activity (Spots Activity and Function form, Cincinnati Knee Rating System). Control participants were excluded if they reported: (1) prior or current ligamentous or meniscal injury or surgery on lower extremities, (2) current or history of pathology or morphology in lower extremities that could cause pain or discomfort during physical activity (contralateral limb; ACLr group), and (3) any pathology or medical condition that may impair their ability to perform the tasks proposed in this study.

**Table 1:** Participants characteristics.

	ACLr (*n* = 13)	CTRL (*n* = 13)
Age (years)	24.6 (9.8)	24.3 (9.2)
Sex	5 M/8 F	5 M/8 F
Height (cm)	1.71 (0.08)	1.71 (0.08)
Weight (kg)	71.66 (9.25)	70.8 (9.05)
Days post-ACLr	115.6 (17.8)	
Graft type (*n*)		
Bone-Patellar Tendon-Bone autograph	8	-
Hamstring autograft	1	-
Allograph	4	-
Physical Activity	95.00 (27.12)	96.15 (12.44)
IKDC overall	58.5 (11.69)	99.5 (1.30)

Values presented as mean (standard deviations) unless otherwise indicated.

### Procedures

Testing took place at the University of Southern California, Division of Biokinesiology and Physical Therapy's Human Performance Laboratory located at the Competitive Athletes Training Zone (CATZ) in Pasadena, CA. All procedures were explained to each participant and informed consent was obtained as approved by the Institutional Review Board of the University of Southern California, Health Sciences Campus. Parental consent and youth assent were obtained for participants under the age of 18 years. After consenting, participants completed the subjective portion of the International Knee Document Committee (IKDC) form and Cincinnati Sports Activity and Function form to determine their current functional status and physical activity prior to injury, respectively. Age, height, weight, dominant limb (defined as leg the participant would kick a ball with), and knee medical history were recorded.

#### Task

**Figure 1:**
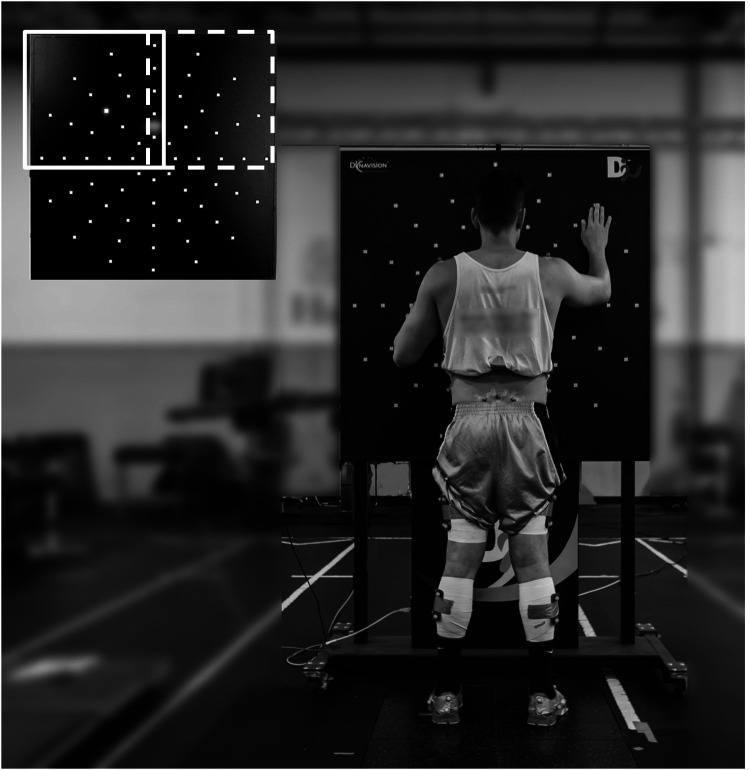
Experimental set-up for the UERT task and the dynavision D2 system. The dimension of the light board is shown at the upper left corner. The white solid square indicates the upper left quadrant and dashed square indicates the upper right quadrant used in the UERT task.

Participants were asked to perform an upper extremity response time (UERT) task under three different attentional conditions. For this task, participants stood on two separate force platforms (BTS P-6000:BTS Bioengineering Corp, Milan, Italy) with their feet shoulders width apart in front of a 4’ × 4’light board (Dynavision D2™ Visuomotor Training device, Dynavision International LLC, West Chester, OH, USA; Figure 1). The light board made up of 64 targets arranged in 5 concentric rings. The board was positioned at a distance in front of the participant so that they were able to reach all the targets on the most peripheral ring without side-to-side trunk movement. The targets were divided in four quadrants consisting of 18 targets each. The top two quadrants were used for the UERT task (Figure 1 upper left corner, solid and dashed rectangles). Participants were instructed to respond as fast as possible to depress a target when it illuminated. After the first illuminated target, each target depressed signaled the illumination of the next target with a latency of 0.02 s. Each UERT trial was performed for a total of 60 s.

#### Attentional conditions

The UERT tasks were performed under three attentional conditions.

Response time only (RT): The response time only condition was introduced first to probe individual's natural loading strategy when performing the UERT task. In this condition, no instructions were given regarding weight bearing and participants were not informed that they were standing on force platform or that ground reaction forces were being recorded. This condition required participants to focus only on response time of the UERT. Prior to testing participants were given the following instructions: “Tap the illuminated targets as fast as you can during the task.” Performance feedback of the UERT task was provided to the participants after each trial. For the next two conditions individual were given two tasks and asked to perform both tasks but prioritize their attention to one of the tasks.

Prioritize limb loading symmetry (LS-RT): Participants were instructed to perform the UERT task while loading their limbs symmetrically. In the LS-RT condition, they were asked to prioritize maintaining limb loading symmetry (LS) while responding to the illuminated targets as fast as possible (RT). They were given the following instructions: “Distribute your weight evenly on the platforms and tap the illuminated targets as fast as you can. In this task, it will be more important that you distribute your weight evenly on the platforms as accurately as possible”.

Prioritize response time (RT-LS): In the RT-LS condition, they were asked to prioritize responding to the illuminated targets as fast as possible while maintaining limb loading symmetry. They were given the following instructions: “Distribute your weight evenly on the platforms and tap the illuminated targets as fast as you can. In this task, it will be more important that you tap the lights as fast as you can.”

**Figure 2:**
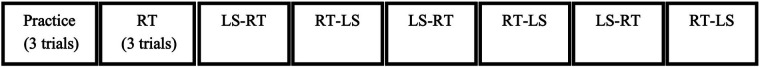
Testing sequence.

After performing three trials in the RT condition, LS-RT was introduced followed by the first RT-LS condition. The second and the third LS-RT and RT-LS trials were then introduced alternatively (Figure 2). To avoid a learning effect, the illuminated targets were presented in a random order for each trial and participants were given 3 practice sessions to familiarize them with the light board. For each condition, 3 trials (60 s each) were used for analysis. Performance feedback of the UERT task was also provided to the participants after each trial in these two conditions.

### Data analysis

Vertical ground reaction force (GRF) and response time were collected during each UERT task. Vertical GRF of each limb was measured using two separate force platforms. Response time, defined as the amount of time from when the light was illuminated to when the light was tapped by the participant, was output from the Dynavision D2™ in seconds to two decimal places.

Vertical GRF impulse was calculated as the area under the vertical ground reaction force time curve during task execution using custom Matlab program (Mathworks, Natrick, MA, USA). Limb loading symmetry (LLS) during each UERT task was calculated as a between limb ratio of vertical ground reaction force impulses using Equation 1. To calculate the LLS in the CTRL group, limbs were matched to the ACLr group based on dominance regardless surgery.

(1) $$\frac{\mathrm{surgical}\;\mathrm{limb}\;(\mathrm{matched}\;\mathrm{limb}\;\mathrm{dominance}\;\mathrm{in}\;\mathrm{CTRL})}{\mathrm{non}-\mathrm{surgical}\;\mathrm{limb}\;(\mathrm{matched}\;\mathrm{limb}\;\mathrm{dominance}\;\mathrm{in}\;\mathrm{CTRL})}\;$$

LLS of 1 indicates equal distribution of weight between the limbs, LLS less than 1 indicates loading of the surgical/matched limb was less that the non-surgical/matched limb; and LLS greater than1 indicates loading of the surgical/matched limb is greater than the non-surgical/matched limb. To determine the degree of limb loading error in each condition, LLS error was calculated as the absolute value of 1-LLS (|1-LLS|) For all conditions, averages of LLS error and response time across 3 trials for each condition were used for analysis.

### Statistical analysis

A priori sample size analyses on primary variables of interests (LLS error and response time) were performed using pilot data collected on 10 subjects (ACLr, *n* = 5 and control, *n* = 5). Data from the pilot study suggested that the results were normally distributed. Sample size calculations for group and prioritization condition comparisons using independent- and paired-samples t-tests indicated that a minimum of 4 participants per group were needed to detect the expected differences in limb loading symmetry between prioritization conditions (Cohen's *d* = 1.85 and power = 0.81) and response time (Cohen's *d* = 2.38 and power = 0.87) in the ACLr group with an alpha level of 0.05.

Separate 2 (Group) × 3 (Prioritization) General Linear Model (GLM) repeated measures analyses were performed to assess the effects of group and focus prioritization on limb loading symmetry and response time. In the case of a significant main effect or interaction, planned comparisons using independent- or paired-samples *t* test were conducted to compare limb loading symmetry and response time between groups and focus conditions. Significance level for all the tests was set at *α* = 0.05 (IBM SPSS Statistics, Version 22, IBM Corp., Chicago, IL).

## Results

### LLS error

**Figure 3:**
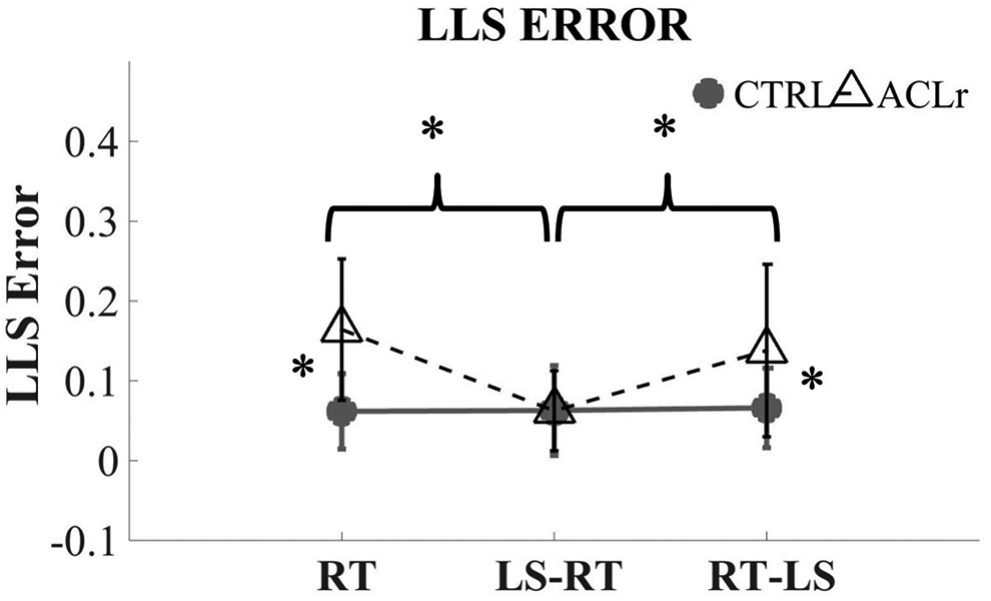
Limb loading symmetry (LSS) error across groups and focus prioritizations.

For LLS error, a significant interaction (*F* = 5.68, *P* = 0.01) between group and prioritization was noted (Figure 3).

When comparing within the ACLr group, LLS error was significantly greater in the RT only condition (*P* = 0.001; Table 2) and RT-LS condition (*P* = 0.01; Table 2) compared to the LS-RT condition. No significant difference was noted among conditions when comparing LLS error within the CTRL group (RT vs. RT-LS, *P* = 0.76; RT vs. LS-RT, *P* = 0.95; RT-LS vs. LS-RT, *P* = 0.82, Table 2).

**Table 2:** The effects of group and prioritization on LLS error, and pairwise comparisons across three focus conditions.

LLS error	ACLr			CTRL			*P*-value		
	RT	LS-RT	RT-LS	RT	LS-RT	RT-LS	Main effect (group)	Main effect (focus condition)	Interaction
Mean	0.164	0.062	0.138	0.061	0.062	0.066	0.014	0.010	0.010^a,b,c,d^
Standard deviation	0.088	0.050	0.108	0.047	0.056	0.049			

^a^ ACLr RT vs. ACLr LS-RT.

^b^ ACLr RT-LS vs. ACLr LS-RT.

^c^ ACLr RT vs. CTRL RT.

^d^ ACLr RT-LS vs. CTRL RT-LS.

When comparing between groups, the ACLr group exhibited significantly greater LLS error in the RT (*P* = 0.001, Table 2) and RT-LS (*P* = 0.04, Table 2) conditions compared to the CTRL group, but not in the LS-RT (*P* = 0.985, Table 2) condition.

### Response time

**Figure 4:**
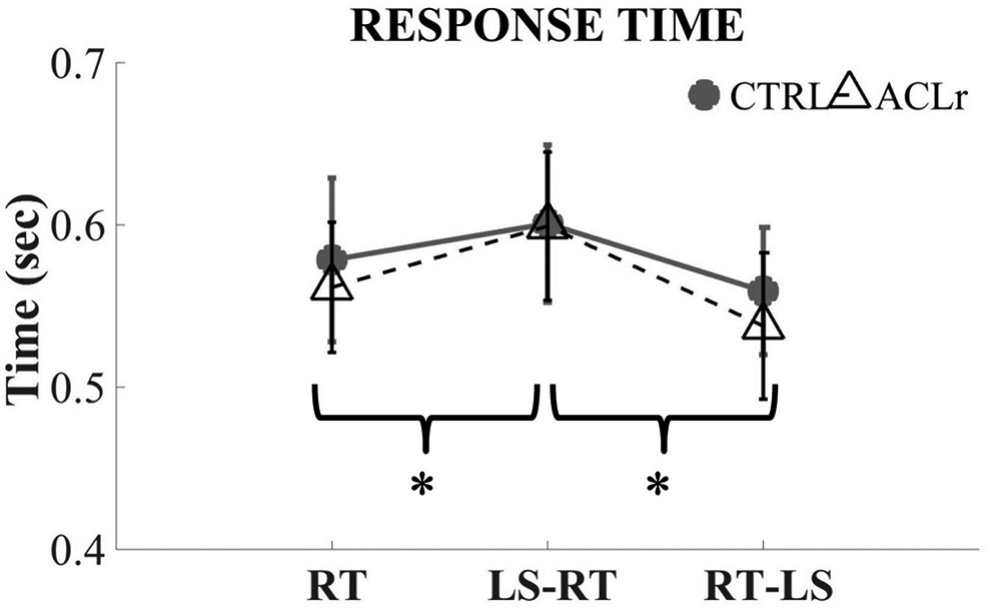
Response time groups and focus prioritizations.

For response time, a main effect of prioritization was observed (*F* = 24.95, *P* < 0.001, Figure 4). When collapsed across group, response time was significantly slower in the LS-RT condition compared to the RT only (*P* < 0.001; Table 3) and to the RT-LS (*P* < 0.001; Table 3) conditions. Response time in the RT-LS condition was not significantly different compared to the RT condition (*P* = 0.469; Table 3).

**Table 3:** The effects of group and prioritization on response time, and pairwise comparisons across three focus conditions.

Response time (seconds)	ACLr			CTRL			*P*-value		
	RT	LS-RT	RT-LS	RT	LS-RT	RT-LS	Main effect (group)	Main effect (focus condition)	Interaction
Mean	0.56	0.60	0.54	0.58	0.60	0.56	0.469	<0.001^a,b^	0.211
Standard deviation	0.04	0.05	0.05	0.05	0.05	0.04			

^a^ RT vs. LS-RT.

^b^ RT-LS vs. LS-RT.

## Discussion

Understanding the extent to which attention prioritization influences limb loading symmetry in individuals following ACLr is particularly important at this stage of recovery. This represents a time in which individuals are re-establishing their loading behaviors in rehabilitation and are increasing their daily activities. As highlighted by previous studies, despite their ability to perform more symmetrically with focused attention, individuals 3 months post-surgery utilize strategies that shift the load away from the surgical limb during tasks that mimic daily activities (7). Using a dual-task paradigm, the current study demonstrates that when compared to non-injured controls, attaining limb loading symmetry may require additional attention in individuals 3 months post-ACLr.

Insight into spontaneous or natural limb loading distribution during standing was provided in the RT condition, as participants were not aware that symmetrical limb loading was a goal or that it was being measured. With explicit focus on performing the UERT task as fast as possible, control participants exhibited relatively symmetrical limb loading with an average of 6% of LLS error. However, LLS error during the RT was 16% in the ACLr group, highlighting a natural tendency to underload their surgical limb. Performance of the UERT test was similar between groups.

The results of the dual-task comparisons suggest that concurrent tasks influence individuals following ACLr differently that non-injured controls. Central processing capacity is limited; during a dual-task condition this capacity is shared between two concurrent tasks (20). If a greater proportion of processing capacity is required by the prioritized task, there is less available capacity to allocate to the concurrent or secondary task. If the secondary task requires more capacity than it is available, performance of that task will degrade. This is seen in the degradation or increase in response time in the dual-task condition (LS-RT) by both groups. In this condition loading symmetrically was prioritized in the instructions. Once participants were asked to focus on loading symmetry and prioritize this goal, performance of the secondary task degraded. Slower response times were observed in the LS-RT compared to the RT condition in both groups suggesting that focusing on loading symmetry depleted the central processing capacity and interfered with UERT performance. However, the fact that participants in both groups demonstrated similar increases response time suggests that the cognitive resources needed to attend to loading symmetry did not differ between groups.

When prioritizing LLS both groups exhibited symmetrical loading with only 6% of LLS error. This is particularly important in the ACLr group, as the single task condition suggests that their natural tendency is to underload their surgical limb. Limb loading error improved from 16% in during the RT to 6% in the LS-RT condition in the ACLr group. These data suggest that improved loading symmetry is achievable when performing a concurrent task if individuals specifically prioritize their loading behaviors.

When individuals prioritized performance of the UERT as fast as possible (RT-LS), response time was similar to the RT only condition in both groups. However, when asked to prioritize response time, loading symmetry degraded in individuals post-ACLr compared to when limb loading was prioritized. On average, 14% error in loading symmetry was observed in the ACLr group compared to 6% in the control group. While the LS-RT condition suggest that focusing on loading symmetry requires additional attention for both groups, the ability to maintain loading symmetry in the RT-LS condition in the controls suggests that loading symmetry may be, to certain extent, an automatic response for non-injured individuals. Automaticity is often conceptualized as the ability to perform a task with minimal cognitive demands and minimal interference from other concurrent information processing (18). The focus on RT did not influence loading symmetry in controls indicating that loading symmetry is a natural or automatic posture that requires minimal cognitive demands. The greater LLS error observed in the ACLr group indicates that achieving loading symmetry is not automatic and requires additional attention that was not being prioritized.

These findings have direct implications for the rehabilitation post-ACLr. Individuals in early recovery may need to prioritize loading symmetry during dual-task training until this posture becomes more automatic. Moreover, the inability to maintain loading symmetry when a concurrent attention is present and prioritized suggests that individuals following ACLr may more readily adopt this underloading strategy during daily activities. During daily activities, individuals often perform more than one task at a time. It is likely that the loading goal emphasized in the rehabilitation sessions may not be carried over as a priority into daily living. As such, the loading practice during daily activities may serve to reinforce the asymmetrical loading strategy which may underlie the persistence of the asymmetrical behavior. Therefore, the present study supports the inclusion of dual- or multi-taking training stimuli during rehabilitation especially during tasks that mimic daily activities.

## Study limitations

Given that standing may be less challenging than other tasks performed throughout the day, the influence of distracted attention on limb loading behaviors may be underestimated in the is study. Interpretation of these data is limited to individuals 10–14 weeks post-ACLr. Self-reported IKDC scores were consistent with those reported in similar cohorts in other studies (18–20), indicating that individuals in the ACLr group were recovering typically. However, it is not known how these results would apply to those who have less typical recoveries. These data do not allow for speculation of how the demands of loading symmetrically change over time. Further work is needed to determine how training can reduce the cognitive demands of loading in the population.

## Conclusion

The present study demonstrates that, in contrast to uninjured controls, maintaining limb loading symmetry during standing is not a natural or spontaneous loading strategy for individuals in early stages of recovery following ACLr. However, when loading symmetry was prioritized, individuals following ACLr are able to achieve typical symmetry demonstrated in healthy individuals. When a second task is introduced and prioritized, this improved symmetry is not maintained indicating that maintaining loading symmetry requires additional attention following ACLr. For controls, the ability to load symmetrically while attending to another task indicates that symmetrical limb loading may be a more automatic response that requires minimal cognitive resources. The present study supports the inclusion of dual- or multi-task training with a focus on loading symmetry during early recovery following ACLr.

## Data Availability

The raw data supporting the conclusions of this article will be made available by the authors, without undue reservation.
